# Perception of Stretch Marks Risk Factors Among Adults in Riyadh, Saudi Arabia

**DOI:** 10.7759/cureus.19561

**Published:** 2021-11-14

**Authors:** Renad A Alageel, Abrar E Bukhari, Amani S Alotaibi, Reeman S Alsalman, Malak M Aldakhilallah, Jihan J Siddiqui, Ibrahim A Al-Omair, Eman A Almukhadeb

**Affiliations:** 1 Dermatology, College of Medicine - Imam Muhammad Ibn Saud Islamic University (IMSIU), Riyadh, SAU; 2 Dermatology, College of Medicine, King Abdulaziz University (KAU), Jeddah, SAU; 3 Dermatology, College of Medicine, King Saud University (KSU), Riyadh, SAU

**Keywords:** striae distensae, risk factors, striae gravidarum, striae cutis, clinical variables, stretch marks

## Abstract

Background

Striae distensae (SD) or stretch marks are a common dermatological condition where the dermis becomes scarred. This phenomenon is associated with many risk factors. This study aimed to evaluate the clinical variables and risk factors in patients with SD in Riyadh, Kingdom of Saudi Arabia (KSA).

Methods

A cross-sectional study was conducted to assess the clinical variables and risk factors for stretch marks among adults in Riyadh, KSA. An electronic questionnaire with an informed consent section was distributed randomly to a sample of adult patients in Riyadh city, KSA, from April to June 2021. A sample size of 512 participants was calculated using the Cochran formula (n = Z2pq/e2), considering 95% confidence and precision of at least ±5%. A 95% confidence level yielded Z values of 1.96, per the normal tables.

Results

A total of 512 participants were included in this study. This study found that 41.4% of the participants were aged 15 to 25 years of which, 70.4% of the participants were females, and 38.2% had a body mass index (BMI) of 24 or more. More than half of the participants (54.0%) indicated white skin color. The most prevalent area of striae, as reported by the participants, was the abdomen (57%). Around 19.1% of the participants with stretch marks stated that they were smokers, and 23.9% stated that they were obese o, had a chronic condition such as hypertension or diabetes mellitus. The majority of the participants (70.5%) had a family history of stretch marks.

Conclusions

This study showed that the prevalence of stretch marks was higher in females, younger participants, smokers, participants with a family history of stretch marks, and participants with a higher BMI and multiparity. More studies should be conducted to assess the correlations between these factors and the presence of stretch marks, and their influence on the quality of life of individuals.

## Introduction

Striae distensae (SD) is a common permanent dermatological condition characterized by scarring of the dermis, which results in linear bands that are initially erythematous to violaceous in color and then fade steadily to become skin-colored or hypopigmented atrophic lines that may be thin or wide [1]. These stretch marks commonly appear on the abdomen, breasts, thighs, and buttocks. Although they do not imply a medical risk, SD may cause esthetic concerns in the affected patients and often present with itching and burning [2]. Stretch marks are visible, long, and narrow scars that develop in areas of dermal damage due to sudden excessive stretching of the skin. They are common in females aged five to 50 years and frequently occur in association with adolescent growth spurts and pregnancy, and also occur in several pathological conditions such as obesity, Marfan and Cushing’s syndromes, and as a result of long-term systemic or topical steroid use. Thus, stretch marks are common and often cause cosmetic morbidity and psychological distress, particularly in women and individuals in certain professions [3]. The etiology of stretch marks is unclear, although various factors have been postulated to contribute to striae formation, including altered hormonal changes, mechanical stretching of the skin, and reduced genetic expression of fibronectin, elastin, and collagen [4]. Striae often develop during adolescence, obesity, pregnancy, endocrinopathies (Cushing’s syndrome), or as an adverse effect of chronic steroidal usage and after surgery.

Striae gravidarum (SG) are longitudinal lesions of the abdomen, breasts, buttocks, hips, and thighs that occur during pregnancy [5]. These show variable coloration ranging from pink, red, and brown. Their prevalence has been reported to range from 50% to 90% in pregnant women in the general population [6,7]. The causes of SG remain uncertain, but they are related to the structural changes that confer skin its tensile strength and elasticity, the reaction force of the skin's collagen string, as well as changes in its flexibility [1,6,8,9]. The prevalence of striae distensae is two and a half times higher in females than in males [10]. African-American women are more severely affected than Caucasian women [11]

Stretch marks are commonly observed in adolescence and affect approximately 30% of individuals during puberty worldwide [12]. Since striae cutis distensae are highly prevalent and have a significant psychosocial impact on the patients’ quality of life [4], in this study, we aimed to evaluate the clinical variables and risk factors in patients with SD in Riyadh, Kingdom of Saudi Arabia (KSA).

## Materials and methods

Study design and population

We conducted a cross-sectional survey from April to June 2021 to evaluate the clinical variables and risk factors in patients with SD in Riyadh, KSA. The study population consisted of adults with stretch marks. The questionnaire was designed in both Arabic and English, and participants had the option of choosing any one language. The study protocol was approved by the Institutional Review Board of College of Medicine, Imam Mohammad Ibn Saud Islamic University (approval No.73-2021).

Sample size and sampling strategy

Upon distribution of our electronic questionnaire, we received responses from 512 participants with all of them having stretch marks (the first question asks if the responder has stretch marks or not, and if they do, they can continue to answer the questionnaire). A sample size of 512 participants was calculated using the Cochran formula (n = Z2pq/e2), considering 95% confidence and precision of at least ±5%. A 95% confidence level yielded Z values of 1.96, per the normal tables.

Data collection

Data were collected through the distribution of the electronic questionnaire to a random sample of adults with stretch marks. Informed consent was obtained from all the participants, and the study was conducted while maintaining maximum privacy, safety, and confidentiality. The study population consisted of adults with stretch marks living in Riyadh, KSA. We excluded adults without stretch marks and those living outside Riyadh. The questionnaire contained questions about sex, age, body mass index (BMI), number of stretch marks, skin color, and color of stretch marks, and the areas most affected by SD. Risk factors were assessed by questions about smoking status, obesity or chronic medical conditions, usage of systemic medications or topical steroids, family history, parity, gestational age at birth, and maternal BMI at birth.

Data analysis

The obtained data were entered into Microsoft Excel software (Redmond, Washington, US) and analyzed using the Statistical Package for Social Science (SPSS) version 20 (BM Corp., Armonk, NY, USA). Categorical variables, including primary variables, were described using frequencies. Univariate analysis for categorical variables was conducted using the Chi-square test to check for all possible risk factors. Logistic regression was used to assess the relationships in the study. The prevalence was determined in percentages with a 95% confidence level. Differences with a P-value < 0.05 were considered significant.

Data management

Data were stored in a safe computer within an encrypted file and were accessed by the research team. Privacy and confidentiality were maintained under all circumstances.

Ethical consideration and issues

For study protocol/study design/methodology, the study was approved by the medical ethics committee of Institutional Review Board of the College of Medicine, Imam Mohammad Ibn Saud Islamic University (ethical approval code: 73-2021).

## Results

A total of 512 participants with stretch marks responded to our questionnaire (see Appendix A for the full questionnaire). We found that 41.4% of the participants were aged 15 to 25 years, while 31.8% were aged 26 to 36 years. Moreover, 70.4% of the participants were females, and 38.2% had a BMI of 24 or more. While 58.4% of the participants indicated few stretch marks, 86.2% reported having white stretch marks. Furthermore, 54% of participants indicated white skin color, while 29.5% reported having a tanned skin color, and 12.6% had brown skin color (Table 1).

**Table 1:** Demographic characteristics of participants, types of stretch marks, and BMI BMI: Body mass index

		Count	N %
Age (years)	15-25	211	41.4%
	26-35	162	31.8%
	36-45	137	26.9%
Sex	Male	151	29.6%
	Female	359	70.4%
BMI (kg/m^2^)	18-20	148	29.0%
	20-22	167	32.7%
	24 and above	195	38.2%
Number of stretch marks	Few	298	58.4%
	Many	212	41.6%
Color of the stretch marks	Red	70	13.8%
	White	439	86.2%
Skin color	Dark brown	20	3.9
	Brown	64	12.6
	Tanned	150	29.5
	White	275	54.0

**Figure 1:**
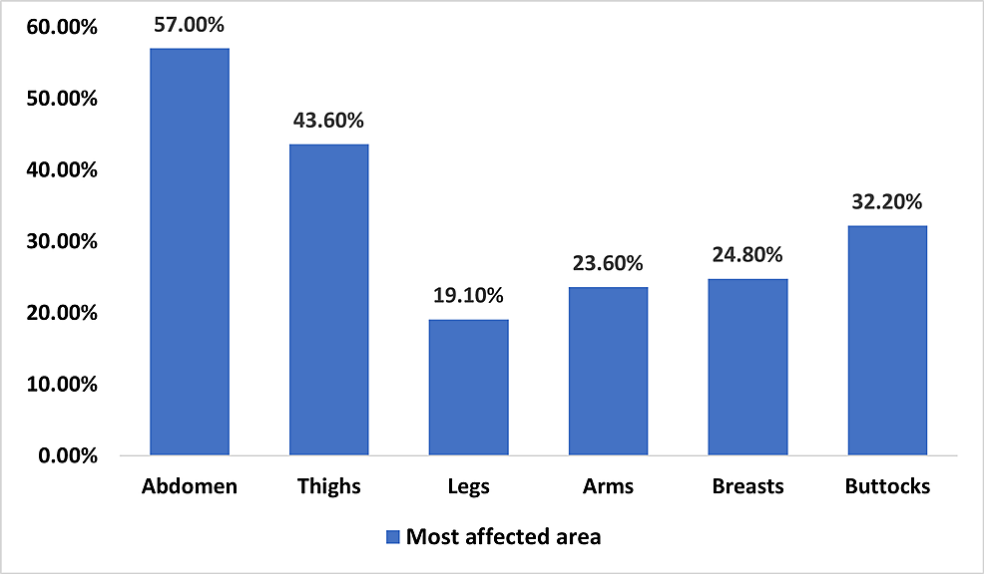
Most affected area

The most prevalent areas of striae, as reported by participants, were the abdomen (57%), thighs (43.6%), buttocks (32.2%), and breasts (24.8%) as seen in Figure 1.

With respect to the medical characteristics, 19.1% of the participants with stretch marks stated that they were smokers, and 23.9% stated that they were obese or had a chronic condition such as hypertension or diabetes mellitus. Moreover, 8.4% of the patients took systemic medication, and 4.3% used topical steroids without medical supervision. In addition, around 12% of the patients reported having skin problems, of which eczema was the most prevalent (58%) followed by vitiligo (22%) and acne (20%). Furthermore, 70.5% of the participants had a family history of stretch marks (Table 2).

**Table 2:** Clinical characteristics of the participants

		Count	N %
Do you smoke?	Yes	97	19.1%
	No	412	80.9%
Do you have obesity or/and chronic disease (hypertension, diabetes mellitus)?	Yes	122	23.9%
	No	388	76.1%
Do you take any systemic medications?	Yes	43	8.4%
	No	466	91.6%
Have you been using topical steroids without medical supervision?	Yes	22	4.3%
	No	487	95.7%
Do you have any skin problems?	Yes	61	12.0%
	No	448	88.0%
If yes, can you write the name of the problem?	Acne	10	20.0%
	Eczema	29	58.0%
	Vitiligo	11	22.0%
Do you have a family history of stretch marks?	Yes	359	70.5%
	No	150	29.5%

**Figure 2:**
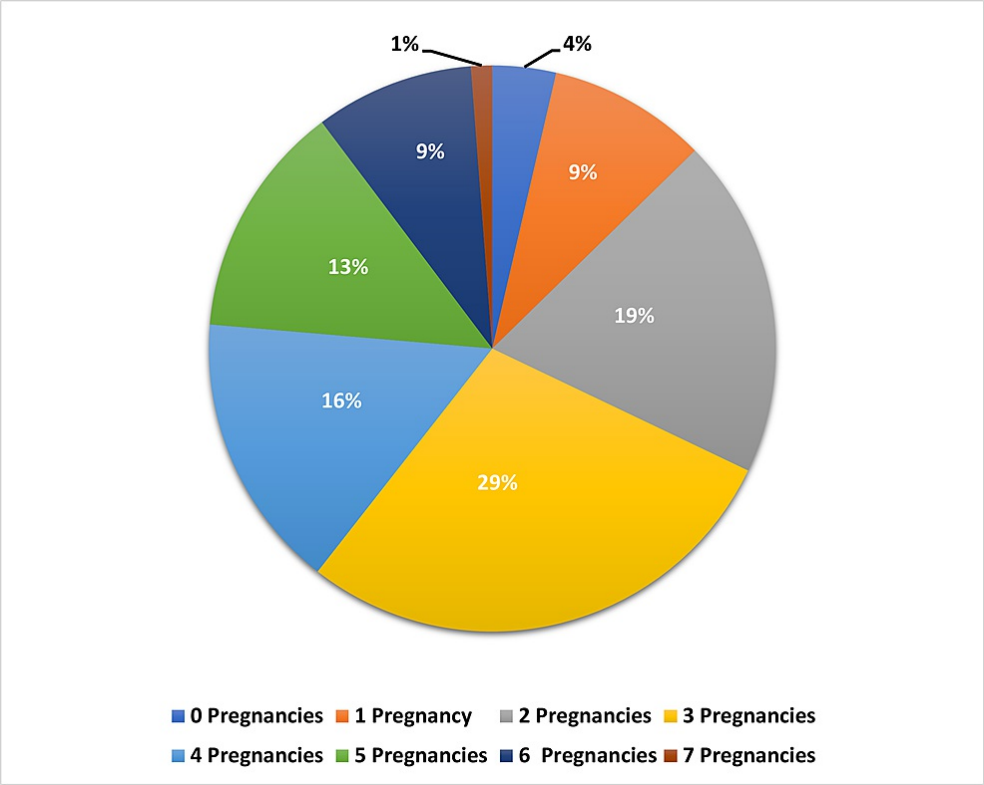
Number of pregnancies (parity)

Among female participants, 29% had three pregnancies, 19% had two pregnancies and 16% had four pregnancies, while 4% had never been pregnant (Figure 2).

Moreover, we found that 45.1%, 41.8%, and 13.2% of pregnant female participants reported a weight gain of 10 kg, 12 to 14 kg, and 15 to 20 kg during pregnancy. Additionally, 55.8% reported a gestational age of 40 weeks at delivery, and 81.6% had a family history of SG (Table 3).

**Table 3:** Clinical characteristics of the female participants

		Count	N %
Average weight gained with pregnancies (kg)	10	82	45.1%
	12-14	76	41.8%
	15-20	24	13.2%
Gestational age at delivery	34 weeks or less	12	7.3%
	36 weeks	20	12.1%
	38 weeks	41	24.8%
	40 weeks	92	55.8%
Family history of striae gravidarum?	Yes	292	81.6%
	No	66	18.4%

## Discussion

Nardelli [13] first described stretch marks as linear scars resulting from the stretching of the skin. These changes occur in all races and have the same clinical appearance regardless of the cause. Clinically, the condition passes through two stages: the first stage is characterized by elevated inflammatory redness (striae Rubra), while the second stage shows white depressions with fine wrinkles (striae alba). Although this health condition is quite common, little is known about its etiology and pathophysiology. Mechanical factors such as the extent and rate of stretching and hormonal factors are the leading causes of stretch marks and are common in adolescence, especially with rapid growth [14].

A previous literature review showed that the prevalence of stretch marks is higher in females than in males across age groups, while Sisson et al. reported that only 39% of males aged 14 to 20 years had stretch marks compared to 61% of females [15]. Additionally, Herxheimer et al. evaluated the incidence of stretch marks in students aged 10 to 16 years and indicated that the percentage of females was 72% [16]. In our study, 70.4% of the responses were provided by females, and striae were frequently seen in younger participants. However, while Kasielska-Trojan et al. did not find a significant age-related difference, some other studies reported results consistent with ours, showing that stretch marks were more frequent in younger individuals [1,8]. Moreover, we found that the most prevalent areas of striae as reported by participants were the abdomen (57%), thighs (43.6%), buttocks (32.2%), and breasts (24.8%). In the study by S Cho et al., the most prevalent areas of striae were the buttocks, lower back, thighs, and calves [14].

Considering the risk factors, we found that 38.2% of the participants with stretch marks had BMI greater than 24 kg/m2. Kasielska-Trojan et al. suggested that BMI is an independent risk factor for the development of stretch marks [17]. This was similar to the results obtained by Thomas et al., who reported that women with higher BMI had more stretch marks [8], and Ogrum A et al. too, obtained matching results [18]. 

Other risk factors found in our study include smoking, the presence of chronic conditions, and a family history of stretch marks. The findings of a study of Osman H et al., who stated that smoking and family history was not related to the presence of stretch marks, did not match our results. Nevertheless, they still reported that women with a positive family history of SG were more likely to develop moderate/severe striae [1]. In contrast, Kasielska-Trojan A et al. reported that the presence of chronic conditions decreased the chances of developing stretch marks five-fold [17].

Among females, a higher number of pregnancies and greater weight gain during pregnancy were associated with the presence of stretch marks. Many researchers found a significant correlation between weight gain during pregnancy, birth weight, and striae formation [1,8,17,19]. Furthermore, women with a family history of SG were shown to be more likely to develop moderate/severe striae than those with no family history of stretch marks [8]. Some authors found that positive family history was strongly associated with SG occurrence, regardless of their intensity. The authors concluded that genetic factors do play a role in the development of SG [7,8,20].

One of the first limitations of this study is that not understanding the questions will result in errors and may affect the results. Furthermore, some participants may not answer the questions honestly and precisely, this will affect the data analysis and may result in bias.

## Conclusions

This study concluded that striae distensae is more prevalent in females, and participants with higher BMI, family history of SG, a higher number of pregnancies, and gestational age of 40 weeks at delivery. More than half of the participants indicated white skin color. Moreover, a strong family history of stretch marks may play a major role in developing the same. More studies should be conducted to assess the correlations of these factors with the presence of stretch marks and the influence of stretch marks on the quality of life of participants.

## Appendices

Appendix A

**Table 4:** Questionnaire

1. Do you have stretch marks?	Yes
	No
2. Age:	15-25
	26-35
	36-45
3. Sex:	Female
	Male
4. BMI:	18-20
	20-22
	24 and above
5. Mid-arm circumference:	
6. Waist-hip ratio:	
7. Number of stretch marks?	Few
	Many
8. Color of the stretch marks?	Red
	White
9. Most prevalent area of striae?	Abdomen
	Thighs
	Legs
	Arms
	Breast
	Buttock
10. Do you smoke?	Yes
	No
11. Do you have obesity or/and chronic disease (hypertension, diabetes mellitus)?	Yes
	No
12. Do you take any systemic medications?	Yes
	No
13. If yes, please write the name of the medication.	
14. Have you been using topical steroids without medical supervision?	Yes
	No
15. If yes, please write the name of the medication.	
16. Do you have a family history of stretch marks?	Yes
	No
17. Do you have any skin problems?	Yes
	No
18. If yes, please share the name of the skin problem.	
19. What is your skin color?	Dark brown
	Brown
	Tanned
	White
20. Number of pregnancies:	
21. Average weight gained with pregnancies:	10 kg
	12-14 kg
	15-20 kg
22. Maternal BMI at pregnancy:	
23. Gestational age at delivery?	34 weeks or less
	36 weeks
	38 weeks
	40 weeks
24. Is there a family history of striae gravidarum?	Yes
	No
